# Combining Structural Connectivity and Response Latencies to Model the Structure of the Visual System

**DOI:** 10.1371/journal.pcbi.1000159

**Published:** 2008-08-29

**Authors:** Michael Capalbo, Eric Postma, Rainer Goebel

**Affiliations:** 1 Department of Cognitive Neuroscience, University of Maastricht, Maastricht, The Netherlands; 2 MICC/Department of Computer Science, University of Maastricht, Maastricht, The Netherlands; University College London, United Kingdom

## Abstract

Several approaches exist to ascertain the connectivity of the brain, and these approaches lead to markedly different topologies, often incompatible with each other. Specifically, recent single-cell recording results seem incompatible with current structural connectivity models. We present a novel method that combines anatomical and temporal constraints to generate biologically plausible connectivity patterns of the visual system of the macaque monkey. Our method takes structural connectivity data from the CoCoMac database and recent single-cell recording data as input and employs an optimization technique to arrive at a new connectivity pattern of the visual system that is in agreement with both types of experimental data. The new connectivity pattern yields a revised model that has fewer levels than current models. In addition, it introduces subcortical–cortical connections. We show that these connections are essential for explaining latency data, are consistent with our current knowledge of the structural connectivity of the visual system, and might explain recent functional imaging results in humans. Furthermore we show that the revised model is not underconstrained like previous models and can be extended to include newer data and other kinds of data. We conclude that the revised model of the connectivity of the visual system reflects current knowledge on the structure and function of the visual system and addresses some of the limitations of previous models.

## Author Summary

Visual perception is very important to us, something we can easily come to realize if we imagine ourselves blind. The visual system consists of numerous interconnected brain areas. If we are to understand the functioning of the visual system, then we will need to understand the *connectivity* between these areas. Current models of the visual system have a number of limitations. One of these is that the *time* it takes for the neural signal to reach a certain area often seems inconsistent with the place of that area in the overall structure of the system; e.g., the signal might arrive relatively quickly at an area generally located “higher” in the visual system and slowly at an area located in the “lower” part. We combine data about the known *connectivity* in the monkey brain with *timing* data to find a network structure that is consistent with both kinds of data. The results show that the timing data can be explained when the network contains direct routes from subcortical areas to “higher” cortical areas. We show that our model has fewer limitations than previous models and might explain unresolved issues in the study of connectivity in the human brain.

## Introduction

**Figure 1:**
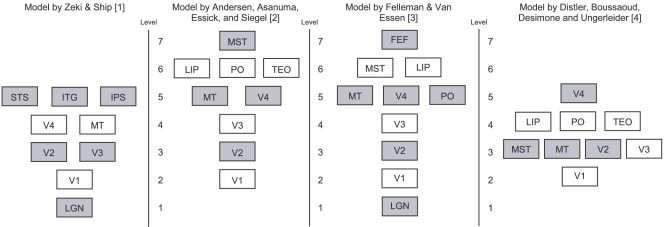
Schematized models of the visual system compared. Areas are placed in a level according to the figures in the original publications. If levels were not present in the original model, areas were placed according to hodology [29], i.e., according to the shortest possible route to connect two areas. The original model by Felleman & Van Essen contains many more levels and areas in the original (see Figure 2).

What are the elements of the visual system in the brain and how are they connected? A large amount of research in vision science has been devoted to these questions. The most apparent way to answer the question of connectivity is to look at the structural connections between functionally defined areas in the visual system, and these have been studied systematically with experimental tracing studies in rats, cats and monkeys. Because of the invasive and time-consuming manner in which these studies have to be done, results are gathered incrementally and scattered across hundreds of separate research publications. If all these separate results are put together, a model of the large-scale structural connectivity of the visual system can be made. For such a model to make sense however, it should be structured according to an organizational principle. Because there are several possible organizational principles, a number of different models can be found in the literature (see, e.g., [1]–[4]). However, the model published by Felleman and Van Essen in 1991 [3] has since been accepted as a standard model, and is cited in numerous academic books (e.g., see [5]) and articles (e.g., see [6]). These models can be compared in Figure 1.

However, a number of limitations of all these models have been identified. We will focus on five limitations, namely that most models are (1) method dependent (2) indeterminate, (3) incomplete, (4) restricted, and (5) invalid with respect to latency data. We discuss each of these limitations in more detail below. (1) *The structure of the models is dependent on the method used.* All three models mentioned earlier are based on studies using different methods. The hierarchical model [3] is based on the pattern of origin and termination of fibers to and from an area, the models by Andersen Asanuma et al. [2] and the model by Distler et al. [4] are based mostly on tracer data and the model by Zeki and Shipp [1] is used to explain segregation and integration of features of the visual image. If we look at Figure 1 we see that all these models differ, yet they all describe the same system. It seems that the structure of these large-scale models is very much dependent on the kind of data used to constrain the model and the phenomena it is designed to explain, whereas these models should agree as they describe the same physical system. (2) *The models are indeterminate.* Hilgetag, O'Neill et al. (1996) developed an algorithm to generate 150,000 candidate hierarchies, all of which agree with the anatomical constraints specified by Felleman & Van Essen [3] in their hierarchical model. Every one of these hierarchies violated fewer constraints than the original Felleman and Van Essen hierarchy. In addition, only two of the considered 30 brain areas were assigned to levels consistently across these candidate hierarchies. From these results, Hilgetag et al. concluded that the constraints (connection types) used by Felleman & Van Essen reduce the number of candidate hierarchies insufficiently. In other words, the hierarchical model, and probably all models like it are under-constrained. (3) *The models are incomplete.* The connectivity data employed by most of the models of the visual system are incomplete because their connection matrix contains many unknown entries [7]. A large number of possible connections have not been investigated. Even with the additional studies done since then, a large percentage remains unknown. (4) *The models are often restricted.* The connection matrices of these models contain only cortico-cortical connections within a single hemisphere. Potentially important connections to and from the contralateral hemisphere and to and from subcortical structures are excluded from the connection matrix [7]. Some of the models in Figure 1 do not even include the LGN. (5) *The models are invalid with respect to latency data.* A final limitation identified by Mountcastle [7] concerns the validity of the models in the light of latency data. According to Mountcastle, single unit recordings show that areas in different levels of the hierarchy of these models are nevertheless simultaneously activated by visual stimuli. Clearly, such simultaneous activation seems to disagree with the associated areas being at different levels in the hierarchy.

These five limitations question the validity of large-scale models of the visual system. Now, more than 20 years after the inception of the first large-scale models, novel insights and experimental results may give rise to a reconsideration of these models. In this paper, we present a method that integrates the anatomical constraints with functional (temporal) constraints extracted from more recent experimental results. Our method leads to the identification of a new structure that meets all the constraints imposed by the integrated data. Before presenting our approach, we expand on the fifth limitation mentioned above, *viz.* the validity of current large-scale models of the visual system in the light of single unit latency recording studies [8],[9]. In our method we will use the data from these studies as temporal constraints for our model.

**Figure 2:**
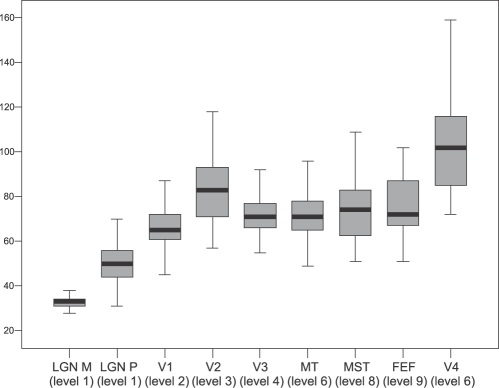
Mean onset latencies in milliseconds (*y*-axis) of several areas in the visual system (*x*-axis). For each area, its level in the hierarchical model [3] is indicated between brackets. Data used from Schmolesky, Wang et al [8].

First-spike time coding might be the only available neural mechanism to bring about rapid behavioral responses, as the alternative neural code, rate coding, is too slow for such responses (e.g., [10],[11]). It has been shown that first spikes can be selective for orientation, faces and optical flow [12]. Given these considerations, an analysis of the spike arrival times at various cortical areas may reveal part of the underlying functional architecture. Schmolesky and colleagues [8] performed a study in which they measured the onset latencies of single-cell responses at several cortical areas in individual anesthetized macaque-monkey brains, evoked by flashing visual stimuli. In Figure 2, we replotted the results obtained by Schmolesky et al. These data reveal a number of inconsistencies with the large-scale anatomical models. For instance we would expect that first spikes arrive later in areas at higher levels in a hierarchy than those in lower levels, but when comparing the latencies in Figure 2 with the models in Figure 1 it turns out that this is often not the case. For example, we can see that area FEF, which is in level 7 in our schematized Figure 1 (it is even at level 9 of the original publication [3]) has latencies comparable to those in V3 (level 3/4), MT (level 3–5) and MST (level 3–7). The latencies of FEF even overlap considerably with those of V1 (level 2). Schmolesky et al. also noted this and they concluded “our data simply indicate that the … anatomical hierarchies fail to account for the initial flow of signals in the visual system and therefore may not accurately represent the ‘functional’ hierarchy of the visual system” (p. 3277). It seems that large-scale anatomical models of the visual system do not agree with the timing data of Schmolesky et al. [8]. Several others have also noted similar inconsistencies. In a review of mammalian spike timing data, Bair [13] observed that neurons assigned to different hierarchical levels are often activated at the same time or in the wrong order with respect to their presumed hierarchical relation. In another review of the available latency data, Nowak and Bullier [14] state that “latencies to visual stimulation in monkey are not ordered as expected from [anatomical hierarchies]” (p. 229) and they cite several studies in support of this claim. The conduction velocity in the efferent fibers also plays a role in latencies, but this alone cannot explain the discrepancies between timing data and structural data fully and determining or approximating this velocity is not possible with our current knowledge.

**Figure 3:**
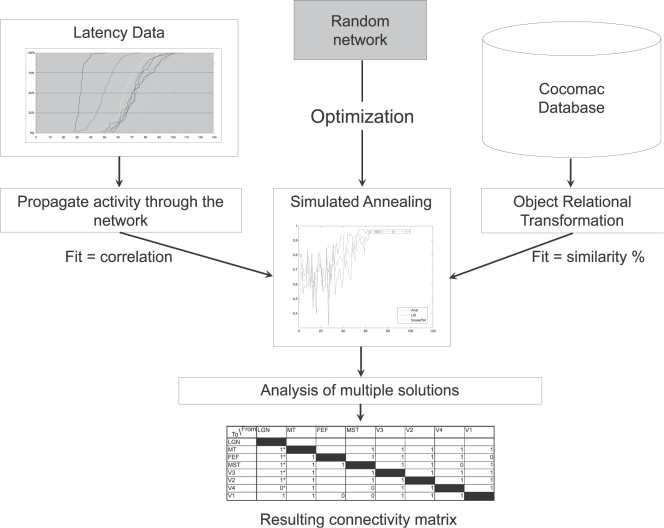
Schematic overview of the method used.

Taken together, it seems that the response latencies in the visual system are incompatible with large-scale models of the known structural connectivity of the visual system. We aim at resolving this inconsistency by integrating structural connectivity data with single unit recording data. Finding networks with a connectivity pattern that fits both kinds of data might not only eliminate the observed inconsistencies, but also suggest a different model better constrained by multiple sources of data. If the model is more constrained, we might learn more about possible connections that have not been investigated yet, or have been excluded from the model. If the generated networks differ substantially (in both fit and architecture) from current models we cannot only conclude that the hierarchical model does not explain the timing data optimally, but we can also point to possible reasons. We want to do this by adopting an extendible method (it should be possible to add both new data and other kinds of data) that can use both structural and functional data as “converging evidence” to model the structure of the visual system. The structural data will be extracted from a database, CoCoMac, which combines several hundred studies about the anatomical connectivity of the adult macaque brain. The functional data will be taken from a single representative study and a review article of the available latency data. To find network topologies that fit both kinds of data an optimization method called simulated annealing will be used. A schematic overview of the method can be seen in Figure 3. In short, with our new method we want to arrive at a revised, more constrained model of the visual system, integrating both structural and functional data, with the additional goal of mitigating the problems of exclusion and incompleteness.

## Results

### Dataset 1

In the first dataset we included the areas used in the study by Schmolesky, Wang et al. [8]. This dataset contains 8 areas: LGN, V1, V2, V3, V4, MT, MST, and FEF. The LGN is the node of the system that receives the first input, the seed node. Note that because we are only studying bottom-up processing, any subcortical area can play exactly the same role as the LGN in the dynamics of the system. Therefore we will call this node SCA (Sub Cortical Area). As we will see later, subcortical areas such as the superior colliculus and pulvinar are also likely candidates for this role. The connectivity data were extracted from the CoCoMac data base [15] in the form of an 8 by 8 connectivity matrix.

**Figure 4:**
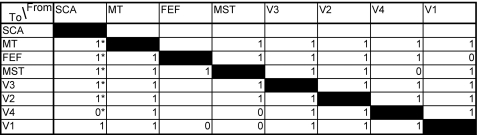
Results for dataset 1. Connectivity entries not in the original connectivity matrix have been marked with an asterisk.

**Figure 5:**
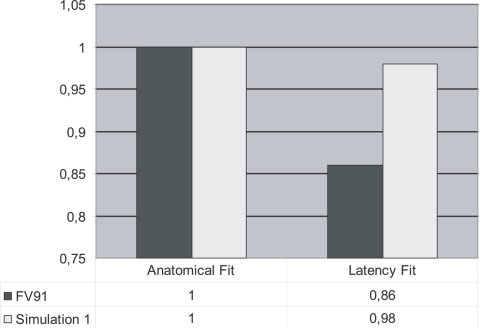
The fit of the hierarchical model by Felleman & Van Essen (1991) compared with the fit of the network generated by our method using dataset 1.

**Figure 6:**
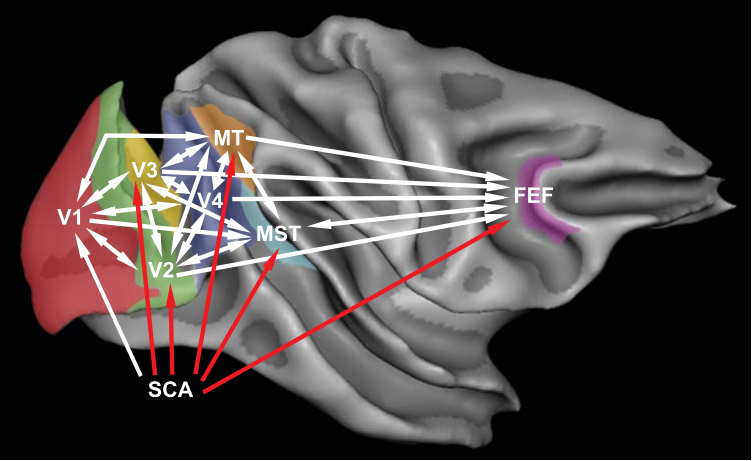
Best-fit connectivity superimposed on an MR-image of the macaque brain. MR-image courtesy of R. Goebel and N. K. Logothetis, rendered with BrainVoyager software. Added connections are red.

The resulting average connection matrix is shown in Figure 4. New connection entries (as compared to the original connection matrix) have been marked with an asterisk. New connections from the SCA have been added to almost every area in the network, except to V4. (The connection to V1 was already present.) In Figure 5 the measures of fit *f*_anat_ and *f*_lat_ of our resulting network are compared to those of the most influential large-scale structural model, hierarchical model [3]. The connectivity and timing costs of the hierarchical model are compared to the costs of the new resulting connectivity matrix. Our network clearly fits the data better. The structural fit (meaning the fit of the network with the known structural data) is maximal (*f*_anat_ = 1) for the solution. This means that our network does not have any connections that are incompatible with the known tracer data. There is an increase in the temporal fit with respect to the hierarchical model, implying that our network fits the available timing data better than the hierarchical model. Simply put: the newly generated network explains the first spike timing data better without violating any constraint from the known tracer data. Figure 6 depicts our solution superimposed on an actual macaque brain. The newly introduced connections are colored. It is clear that a subcortical route has been added to accommodate the temporal constraints. As we shall argue in the discussion this finding confirms Mountcastle's (1998) suspicion that subcortical routes play a significant role in cortical connectivity and agrees with recent connectivity studies.

### Dataset 2

**Figure 7:**
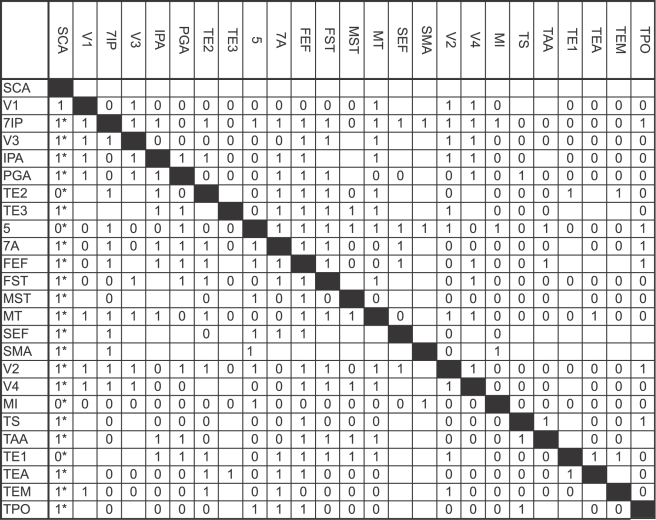
Results for dataset 2. Connectivity entries not in the original connectivity matrix have been marked with an asterisk.

**Figure 8:**
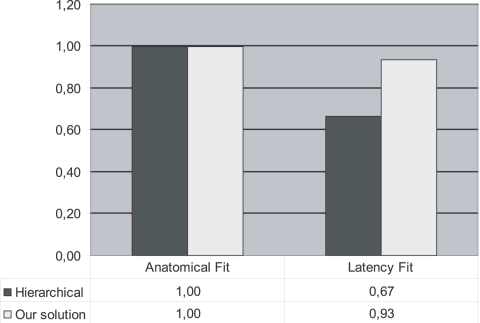
Fit of the hierarchical model by Felleman & Van Essen (1991) compared with the network generated by our method using the dataset 2. Results graphed in the same manner as in Figure 5.

In our results with dataset 1 we have shown that the method works for a small dataset from one parcellation scheme. With dataset 2 we want to use our method on a larger dataset, with more areas from different parcellation schemes (using ORT, [16]), hoping to increase the validity of our claims. Also, a larger dataset might increase the constraints on the model. The first spike time data chosen for this purpose were those of Lamme & Roelfsema [12]. After applying the inclusion criteria, we used the data for the following 27 areas: SCA, V1, 7ip, V3, Ipa, Pga, TE2, TE3, 5, 7a, FEF, FST, MST, MT, SEF, SMA, V2, V4, MI, TS, TAa, TE1, TEa, Tem, and TPO. The resulting connection matrix can be seen in Figure 7. New cell entries that differ from the original connectivity matrix have been marked. The connections added to the matrix as a result of including the constraints of the timing data are again mainly connections from the Sub-Cortical Areas (SCA). New cell values also show that some connections have to be absent in order to account for the timing data. In Figure 8 the fits of the most prominent large-scale model, the hierarchical model [3], and our network are compared. Again, the connectivity and timing costs of the hierarchical model are compared to the costs of the new resulting connectivity matrix. The anatomical fit is at its maximum value of 1, meaning that the resulting network is in complete concordance with the known anatomical data. Our network is clearly an improvement over the hierarchical model regarding the fit with the latency data (an increase from 0.67 to 0.93). These results indicate that adding sub-cortical routes to the network yields a substantial increase of the timing fit. These results establish that the inclusion of the subcortical routes is also essential for explaining latencies in large visual networks.

**Figure 9:**
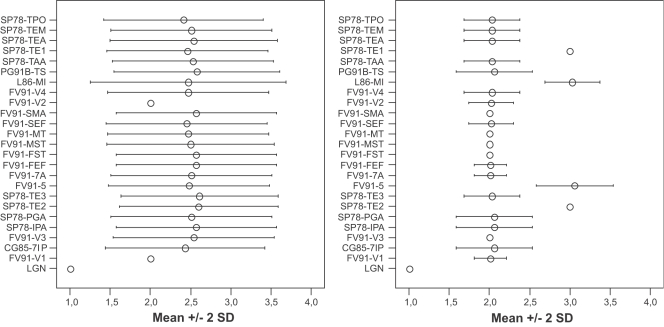
Comparison of the distribution of areas (*y*-axis) over the levels in the hierarchy (*x*-axis) in 100 solutions. On the *x*-axis is the average level an area is assigned to +/− two standard deviations. On the left side the distribution is given without the areas being constrained by the latency data. On the right the distribution is given when the timing constraints are used. The areas are indicated by their respective area names and parcellation schemes (as indicated in CoCoMac) separated by a hyphen.

As mentioned in the introduction, one of the shortcomings of the large-scale structural connectivity models is their indeterminate organization, most notably the indeterminate organization of the hierarchical model [17]. Our method integrates two types of constraints, thereby reducing the “indeterminateness”. To confirm the reduction of indeterminateness, 100 solutions were analyzed to determine the levels at which individual areas were placed. Subsequently, we determinen the average level (and variance) of each area. Figure 9 shows the results of this analysis. In the left-most part of Figure 9 we can see that when using our method with anatomical constraints only, we get roughly the same indeterminate organization as observed for the hierarchical model, even though we used a different cost function than was used in the original publication. Areas can be at multiple levels without violating the anatomical constraints as Hilgetag, O'Neill et al. [17] have pointed out. The right part of Figure 9 shows the results for the combined temporal and anatomical constraints. The number of candidate hierarchies is reduced considerably. More areas are confined to a single level of the hierarchy, and the remaining areas have a smaller number of possible levels to which they can be assigned. Overall the determinateness (or constrained-ness) is greatly improved due to the inclusion of timing data.

## Discussion

This paper presented a data-driven method to generate plausible models of cortical connectivity between areas of the visual system based on a combination of anatomical and temporal data. Our results show that connectivity patterns with subcortical routes fit the timing data of the visual system better then those without subcortical routes, such as the hierarchical model [3].

### Advantages of the Model

**Figure 10:**
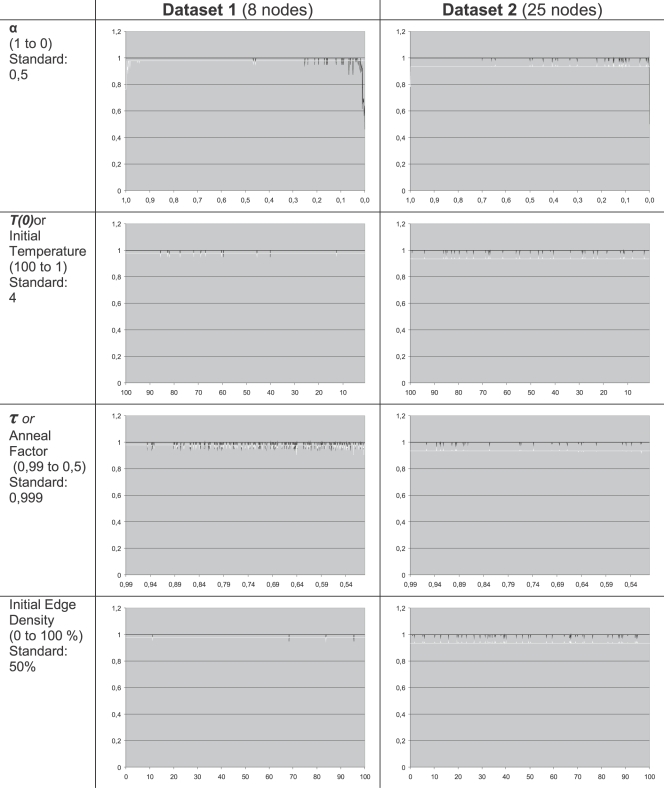
Illustration of the parameter sensitivity of the model. Each graph shows the anatomical and latency fits (vertical axis) as a function of the parameter value. The varied parameter, the range of variation and the parameter actually used in the results are listed in the first column, the total fit for the first dataset is plotted in the second column and the fitness for the second dataset is plotted in the last column. Within each plot, the black line represents the anatomical fit and the white line represents the latency fit.

One of the strong points of the method used is that it is completely data driven. Large-scale network architectures can be generated with one main assumption only: the time it takes for a signal to pass from one area through the afferent pathway to another area can be treated as a unitary whole. We do not need to rely on any assumptions about the structure or the hierarchy of the areas in the network. One of the possible pitfalls of neural networks models is that the complexity of the model does not add to the predictive power of the model. Sometimes models require setting many parameter-values of which the validity is hard to ascertain. In our method there are only four annealing parameters that need to be set to perform an analysis, the effect of these parameters are well-known [18] and the results do not depend critically on the specific parameter settings, as we can see in Figure 10. See Methods, Optimization, for more details.

### Possible Additional Constraints

Our method combined two types of constraints derived from connectivity and timing data, which is more than the single type of constraint used in earlier work [1],[3],[19]. One might argue that more than two types of constraints may or need to be included to generate even more plausible models of cortical connectivity. We mention three additional types of constraints: (1) the conduction velocity, (2) the inter-area distance and, (3) variance in spike time latencies. Although the inclusion of additional types of constraints is rather straightforward in our method, we also motivate why we did not include them in our method.

1. *Conduction velocity*. One might argue that conduction velocity is a possible additional constraint that needs to be added in our method, because it affects the first spike latencies. For instance, the relatively early response of area FEF could be due to a high conduction velocity in the fibers leading to FEF, rather then due to the existence of a direct route to FEF. We argue that it is not beneficial to add conduction velocity as a constraint to our method for three reasons; (I) it cannot explain the latency data fully, (II) data on cortico-cortical conduction velocities are scarce, and (III) conduction velocity cannot be reliably estimated. We will elaborate briefly on these reasons. (I) *Differences in conduction velocities cannot fully account for the inter-area latency differences*. In the hierarchical model [3] for instance, areas separated by one level or more have extremely small differences in first-spike latencies (e.g., MST – FEF differ in 1 level and 1 ms in latency; V1 – MT differ in 4 levels and 5 ms in latency, whereas V1-V2 differ in 1 level but 18 ms in latency) and sometimes the latencies are even reversed (e.g., V4 – FEF). This also holds when the hierarchy is based on the shortest possible route to connect two areas like the model used by Petroni, Panzeri et al. [20]. (II) *Relatively little is known about conduction velocities of cortico-cortical connections* [21]. Most of our knowledge on conduction velocities stems from neural cells outside of the cortex and was not investigated in mammals. This limitation is aggravated by the fact that conduction velocities differ substantially between cortical and subcortical pathways [22] and species (cf., e.g., compare Nowak, James et al. [21] with Girard, Hupe et al. [23]). (III) *The conduction velocity cannot be reliably estimated*. One might argue that even if the actual conduction velocities between areas have not been measured, they may be generalized from an estimation of the conduction velocities in an entire functional stream (e.g., the magno- and parvocellular streams). We argue that knowledge of the conduction velocity of different cell types in different functional streams does not automatically lead to a reliable estimate of the conduction velocity. Conduction velocity is dependent on myelinization and the diameter of neurons and these are known to differ for different cell types. Different cell-types are associated with different functional streams and therefore conduction velocity might differ per functional stream. However, the number and nature of functional streams in the visual system and their constituent cell types are debated (e.g., see [24]–[27]). It is unclear how the conduction velocity of connections between areas within one functional stream or between two functional streams could be estimated. The fuzzy demarcation of the functional streams and the lack of knowledge about how the conduction velocities of different cell types “add up”, make any estimation quite unreliable. Despite the current limited knowledge on conduction velocities, it would be interesting to study the effect of conduction velocities by systematically varying conduction velocities in a model where all else is constant and we plan on doing this in future work. To sum up, conduction velocity probably does play a role in latencies, but we did not add it as a constraint because it cannot explain the discrepancies between timing data and structural data fully, data on cortico-cortical conduction velocities is scarce, and any approximation of the conduction velocity in a functional stream is unreliable.
2. *Inter-area distance*. Another constraint that might influence the spike timing is the distance between areas. It is important to note that the Euclidean distance between areas represent the lower bound on the true inter area distance. Actual inter-area fibers must follow the sulci and the gyri, in effect increasing their lengths beyond the Euclidian distance. To the best of our knowledge, actual inter-area fiber distances are not known. However, Kaiser and Hilgetag [28] have shown that only a small percentage of the connections in the macaque brain are curved so that Euclidean distances might be a good approximation of true inter-area distances. But even when trying to ascertain true inter-area distances some problems remain. One of these problems is the use of Objective Relational Transformation (ORT) [16] as is done for dataset 2. ORT transforms multiple areas from multiple parcellation schemes into areas in a single coordinate free scheme. As a consequence, information on absolute distances is lost. Therefore, the inter-area distances for dataset 2 cannot be determined. Another problem is that, for reasons mentioned above in the context of conduction velocities, inter-area distance *differences* probably do influence timing, but not to the extent that it can explain latency differences. Overall, inter-area distance was not used as an additional constraint because true distances are not yet known, measuring distances between areas from different parcellation schemes is problematic and it is unlikely that inter-area distance differences could explain large latency differences.
3. *Variance*. Latencies vary across multiple replications under the same experimental conditions. In our method, the simulated spike times are deterministic and fall within discretized intervals. Given an average latency, the variance represents a measure of certainty on the spike times. Larger variance implies more uncertainty about the actual latency and relaxes the associated timing constraint. Variance might have been incorporated as an extra term in the evaluation function for dataset 1 (for which variances are available), but not for dataset 2. The data in this set are obtained from a meta analysis of from multiple studies [12]. The raw data from the original studies used in the meta-analysis or data from another review paper (e.g., [14]) might have been used here, but then we would also have lost the advantages of weighing the data [see 12].

### Resolving Inconsistencies

**Figure 11:**
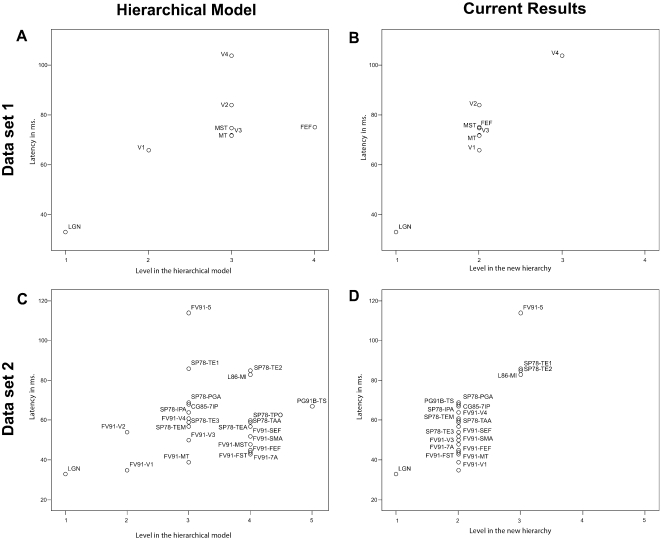
Comparing the hierarchical model [3] with the networks generated by our method, with regard to latency. On the *x*-axis is the level in the hierarchy an area is in when only considering the shortest possible route from the source node. On the *y*-axis is the first spike latency in ms. In part A and D the level structure of the hierarchical model can be seen, in part B and D the same is plotted for the structure suggested by our results. Area names (indicated as in Figure 9) are included for general reference, overlapping labels omitted for clarity. Areas in a higher level in a hierarchy should have longer latencies then areas lower in the hierarchy.

Do our results really solve the inconsistencies between the latency data and the earlier anatomical models? Clearly, if a model agrees with latency data, all the areas assigned to higher levels of the hierarchy should have longer latencies then those assigned to the lower levels. In Figure 11 the timing data is plotted as a function of the level an area is in. This is done for the hierarchical model [3] (Figure 11A and 11C) and for our network (Figure 11B and 11D). In Figure 11A and 11C the sequence of the first spikes does not follow the levels, once again illustrating the shortcomings of all earlier large-scale models. For instance, in Figure 11A, FEF and V4 pop-out as areas with “wrong” timing for their position in the hierarchy. Figure 11B and 11D show that the networks generated by our method do not suffer from this shortcoming; each area is assigned to its appropriate temporal level. As is evident from Figure 11B and 11D, our solution has three levels only.

### Alternate Explanation of the Latencies

We have argued that our results solve the inconsistencies between the latency data and earlier anatomical models. An earlier study has attempted to show that these inconsistencies do not exist [20]. Petroni, Panzeri et al. simulated the cortical network of the areas examined by Schmolesky et al. [8], using the connectivity matrix of the hierarchical model [3]. However, Petroni, Panzeri et al. [20] used something they call the hodology [29], i.e., the shortest possible route to connect two areas, to determine the level at which an area is placed, instead of the levels of the earlier models [1]–[4]. Their simulated latencies resemble the real latencies. They conclude that hodology correlates better with latency than the hierarchical organization. However, our work shows that even the hodological hierarchy can not explain the latencies fully. In the results of Petroni, Panzeri et al. [20] it is already evident that the timing in areas FEF and V4, prominent dorsal and ventral areas, do not fit the model. In their own words: “two areas where we found some disagreement between simulated and experimental latency were FEF … and V4”. Furthermore, as we have argued earlier, even a hierarchy based on hodology cannot explain the extremely short latencies or :reversals” in the latencies. Our results show that the hodological hierarchy cannot explain the overall latencies optimally. In Figure 11 we have shown that even when hodology is considered (the topology we use is always based on the shortest route) the hierarchical model remains incompatible with the latencies. We shall argue below that our explanation provides a better alternative, because it addresses some of the limitations of previous models and might explain some functional characteristics of the human visual system.

### The Importance of Subcortical Connections

For both our datasets, the most prominent and remarkable result is the large increase in goodness of fit with the latency resulting from the introduction of direct connections from the subcortical areas (SCA) to the cortical areas, bypassing V1.

Such direct connections are typical long-distance projections. It has been suggested that the brain shows strict optimal component placement and therefore only has short projections between adjacent brain areas. However Kaiser and Hilgetag [28] noted that long-range projections also exist and that they have an essential role to play, e.g. in the minimization of processing steps. Our findings demonstrate this principle perfectly.

Additional analysis reveals that discarding all subcortical areas from the dataset, the best fit is to connect all extrastriate areas to V1, with the exception of V4. Although the resulting connectivity pattern is still superior in terms of fitness to the hierarchical model (i.e., *f*_anat_ = 0.947, *f*_lat_ = 0.871) the fits are still smaller than those obtained in our main solution. Apparently subcortical pathways explain the data better then cortico-cortical “shortcuts”.

The importance of the subcortical route has been recognized before in various publications. Lamme and Roelfsema [12] noted that a reason for the lack of correspondence between the hierarchical organization and the response latencies might be that subcortical structures like the LGN, the superior colliculus (SC), and the pulvinar (PUL), also project to various extrastriate areas. Even Petroni et al. [20], who claim there is no large discrepancy between latencies and the hierarchical model, propose that the extremely fast response in FEF might be caused by a subcortical connection through the superior colliculus. It is known that not only the LGN, but also the PUL and the SC have retinal inputs [30]–[32], making direct connections from the cortex to these areas true shortcuts from the retina.

With the additional help of the CoCoMac database [15] and Objective Relational Translation (ORT) analysis [16], a literature search was done to assess the connections from the subcortical areas suggested above, to the cortical visual areas named in our results. Except for the obvious connections to V1, the LGN is also connected to V2 [33]. The pulvinar is a structure that is densely connected to cortical areas, i.e., it is connected to V2 [34], V3 [35], V4 [36], MST [37] FEF [38], and MT [39]. Interestingly, MT not only resembles an early visual area because of this subcortical connection but it is also an early visual area in the way that it matures [6]. Although earlier studies repeatedly suggested the SC might be connected directly to the visual cortex [20],[40], little structural evidence for the existence for such connections was found. Relatively few studies have addressed the presence of these connections. A connection from the SC to V1 exists [41], but connections to the following visual areas were examined but not found: V2 [42], V3 [43], PO [34], IT [44]. A possibility is that the SC is involved indirectly by feeding input to the pulvinar [45] or that it is connected via the mediodorsal thalamus [46]. Summarizing, the subcortical routes suggested by our results are in line with the above findings as all “new” subcortical connections (connections from SCA in the figures) found in the results from dataset 1 have been confirmed by anatomical research. These routes run through the lateral geniculate nucleus and the pulvinar (but not through the superior colliculus). The fact that the predictions made by our method have been verified validates our method and lends credibility to our results.

Subcortical pathways appear to play a larger role in the propagation of the visual signal then was assumed before. More specifically, the connections from subcortical areas to extrastriate areas have been left out of most models of the visual system. Therefore these connections need to be considered in any future description of the structure of the visual system.

### Towards a New Model

**Figure 12:**
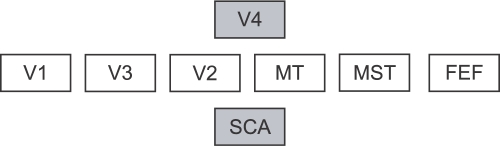
Hierarchies of the visual system compared. Hierarchy proposed in this article with the areas from dataset 1.

Because of all the reasons described above we propose a new, revised organization of areas that is based on both timing and structural data (see Figure 12). It is important to note that the organization proposed here is based on two kinds of data only, and as such only reflects the combination of first spikes and structural connectivity. Therefore, it can not claim to explain other aspects of hierarchy like the increasing complexity of visual responses or receptive field size. In how far the organization we propose reflects the “general organizational principle” (if such a thing exists at all) might be dependent on the importance of the processing of first spikes compared to all processing in the cortex. It might well be that the inclusion of other kinds of data (e.g., receptive field size) will reorder the organization. Another possibility is that in order to explain different kinds of data, different organizations are necessary, and no single organization exists that can explain all aspects of the visual system.

### Human Visual System

Our study is entirely based on connectivity and timing data from the macaque visual system. What does our work tell us about the structure of the *human* visual system? Although the homologies between monkey and human visual cortex remain uncertain for some areas, one of the main reasons for studying the monkey visual cortex are the clear similarities with the human visual cortex [47]. All the areas from dataset 1 have a more or less clear homology in the human brain [48],[49] enabling at least some generalization from our results to the human visual system.

Our results, when generalized to the human brain, might explain some recent findings in humans. Goebel et al. [50] looked at the functioning of the dorsal and the ventral stream in two blindsight patients with long-standing post-geniculate lesions (FS and GY). These patients show close to normal brain activity in hMT+ and V4 although a large part of V1 has been destroyed. Similar results were found in a patient with hemianopia in the entire right visual field, who could still report movement and color change in his blind hemifield (Riddich syndrome). fMRI activity was reported in V4/V8 and V5 in the lesioned hemisphere and MEG recording showed it preceded V2/V3 activity [51]. A functional connectivity study showed that there was a flow of information from V5 to V4 and V2 [52]. How can areas higher “upstream” in the visual system be activated normally when almost all of their input from lower levels has been cut? Subcortical pathways like the ones suggested by our simulations, originating in LGN and the pulvinar might play an important role in explaining the residual functioning of the brain in blindsight and Riddich syndrome.

### Future Work

To further understand the structure of the visual system, future work should include attempts to complete the subcortical pathways in the connectivity matrices. This goal might be reached by adding existing tracer studies to the CoCoMac connectivity database [15] or by doing new tracer studies into subcortical pathways. It would also be very helpful for our understanding of the large-scale structure of the visual system to see more latency studies like the one done by Schmolesky et al. [8] with more areas. This would allow us to use the current method without having to rely on the “averaging” methods currently used in dataset 2 [from 12]. Our current approach could also be aided by more research into conduction velocities in the cerebral cortex, the exact role of conduction velocities in explaining latency data remains an open question. A modeling study similar to this one using conduction velocity as an (extra) constraint might help to resolve this question.

### Conclusion

By combining data from both structural connectivity and spike timing experiments using a data driven method with few assumptions and parameters, topologies that fit both kinds of data have been found. The results show the necessity of subcortical routes to explain spike-timing data. Review of the literature demonstrates that most of the connections predicted by our method appear to exist. Furthermore we show that we are able to further constrain our model, in effect reducing the problem of indeterminacy associated with previous models [17]. We conclude that our method successfully incorporates structural and functional data to arrive at a new large-scale model of the visual system that underscores the importance of subcortical routes.

## Methods

Our method employs the following procedure: we define a connection matrix W in which each element *w*_AB_ represents the presence (*w*_AB_ = 1) or absence (*w*_AB_ = 0) of a connection from area A to area B. Initially, the elements of the connection matrix are randomly assigned a value such that half of the elements is set to 1 and the remaining elements is set to 0. Subsequently, the values in the connectivity matrix are adapted using an optimization algorithm in order to minimize violations of the anatomical and functional constraints. The optimized matrix represents a connectivity pattern, i.e., a model for the connectivity of the visual system that is in agreement with the anatomical and temporal data. In what follows we describe the acquisition of the anatomical and temporal data, the criteria for data inclusion, the evaluation of candidate connectivity patterns, and the optimization algorithm.

### Anatomical Data Acquisition

Anatomical connectivity data was obtained from the CoCoMac (“Collation of Connectivity data on the Macaque brain”) database [53]. At the moment of writing it contains the details of more then 400 studies about the anatomical connectivity of the adult macaque brain using tracer studies. CoCoMac represents all of this data in an objective, coordinate-free, parcellation-based fashion and enables the user to integrate contradictory findings in the literature, depending on the choice of several parameters. The advantage of using CoCoMac over data from individual studies is that it combines hundreds of tracer studies into a single connectivity matrix. With a mathematical method called ORT (Objective Relational Transformation), it is possible to combine and transform brain mapping data from any parcellation scheme to a coordinate-independent freely chosen parcellation scheme [16]. This allows us to combine connectivity data on areas from several parcellation schemes in one of our datasets (dataset 2). The database is publicly available and can be queried through the online interface CoCoMac-Online at http://www.cocomac.org/ [15].

### Timing Data Acquisition

We define two datasets for the timing data. The first dataset is from a single study mentioned in the introduction [8]. The data was collected from four monkeys, over a relatively large number of recording units (558) in nine areas of the brain, measured repeatedly and with a broad range of visual stimuli designed to elicit a response from the entire visual system. We obtained part of the original data from the authors and therefore we were also able to determine the variance in the data. The second dataset is from a review which collected data from multiple studies ([12], box 2, p 573). It therefore includes data on more areas then the first dataset. It suits our purposes very well, as it not only includes several studies but it also weighs them as to reflect the reliability of each experimental finding.

Comparisons of timing data in the literature often suffer from differences in experimental and analytic methodologies between studies. Although the first dataset is the smaller of the two, it does not suffer from these “incompatibility effects”, because all measurements were made within individual monkeys using common stimulus presentation and analysis techniques. This is especially important as we are interested in differences *between* latencies across the visual system. The second dataset is larger than the first, allowing us to determine if the results can be generalized to larger systems. However, the data in the second dataset are probably more prone to “incompatibility effects” because they come from a more varied set of experiments and experimental conditions.

### Data Inclusion Criteria

For each of the two datasets, two conditions needed to be satisfied before the data belonging to a particular brain area could be included: (1) Both first spike data and connectivity data are available. For instance, the connectivity data for Ts, Ts1, Ts2, and Ts3 were available whereas the functional data were only available for Ts. Therefore only Ts was included in the analysis. (2) Areas included in the selection should be considered part of the visual system and should not be too large to be useful in the analysis. For instance, area PreFr (prefrontal) from Lamme and Roelfsema [12] was excluded because it contains a very large number of other areas and because it is arguably not part of the visual system. Because the exact mapping relation between areas FEF and 8a is controversial ([3],e.g., compare [54],[55] we decided to exclude 8a to resolve any uncertainties.

### Evaluation of Candidate Connectivity Patterns

In order to be able to optimize the connectivity matrix, we need a numerical measure of the goodness of fit of timing and connectivity data for any given connectivity pattern. This allows us to search for the best fitting network and is essential for the used optimization technique employed. Below, we define measures for the anatomical fit and temporal fit and combine them into a single overall fitness measure.

The definition of the anatomical fit is relatively straightforward. We define the anatomical fit *f*_anat_ on the unit interval as the proportion of corresponding connections between a candidate connection matrix (CM_A_) and the anatomical connection matrix retrieved from CoCoMac (CM_B_), i.e.:
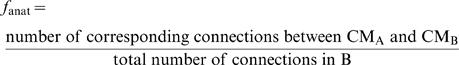



When *f*_anat_ is 0 there are no corresponding connections between A and B (worst fit), and when *f*_anat_ is 1 both are in complete agreement (best fit). Note that the fit will decrease when a connection that is established absent in CM_B_ is present in CM_A_, but the fit will be unaffected when a connection that is empty CM_B_ is present in CM_A_. This implies that violating an established absent connection constraint is treated identical to violating an established existing connection.

The temporal (latency) fit, *f*_lat_, is defined as a linearly transformed Pearson product-moment correlation coefficient (PMCC). It expresses the similarity between (the order and magnitude of) the timing data and our simulated timing data (see below). The function *f_lat_* should provide an indication of the degree to which the orders of activation of nodes in two networks agree. Although rank correlation seems more appropriate for the simulated timing data because they contain ordinals, the use of rank correlation would mean losing all sensitivity to distances between latencies and therefore PMCC is used because it accommodates the continuous values of the real timing data, which is measured at the ratio level. The minimum value of the PMCC is −1 (worst fit) and the maximum is 1 (best fit). Because *f*_anat_ has a range of 0 to 1 and we want *f*_lat_ to have the same range we apply a linear transformation so that:
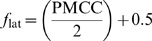



In order to simulate the timing data for our candidate connection patterns, we employ a version of the “shortest path” algorithm [56]. We start by determining the node that receives the initial input (the seed node). This node is defined as being active at the first time step (*t* = 1). We then use the following function to propagate activity through the network for *t*>1:
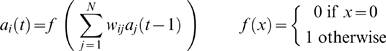
where *a_i_*(*t*) represents the activity of node *i* at time *t* and *w_ij_* the element of the connection matrix, i.e., the connection from node i to node *j* representing the presence (*w_ij_* = 1) or absence (*w_ij_* = 0) of a connection. Nodes connected to an active node will be active the next time-step and will remain active afterwards (*w_ii_* = 1, for all *i*). If all nodes of the network are active, each node is assigned a level equal to the number of connection steps from the seed node. In all simulations, the number of time steps is sufficiently large to allow activations to propagate through the entire network.

The overall fit function *F* is defined as the weighted sum of both fitness measures, i.e.:
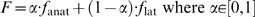



The two measures of fit are weighted by factor *α*.

### Optimization Algorithm

The optimization algorithm used in our method is simulated annealing. Simulated annealing is a stochastic combinatorial optimization algorithm belonging to the class of methods known as gradient descent algorithms. What makes it especially suited for our needs is that it is capable of finding solutions that obey multiple types of constraints. In addition it is generic in that it does not require an explicit knowledge description of the problem at hand [57]. The simulated annealing algorithm was first described by Kirkpatrick, Gelatt et al. [18] and has been applied before for optimizing features of cortical networks [28]. In our method, the algorithm makes changes to individual elements of the connection matrix by applying the rule that each change should increase the fitness. The algorithm varies the strictness with which the rule is applied. Initially, the degree of randomness (or temperature *T*) is high, meaning that changes that decrease the fitness are also allowed, and the values of individual elements are updated at random. As time progresses, the degree of randomness is lowered towards full determinism (i.e., zero temperature). At this stage, connections are updated by applying the rule strictly. The quality of the final solution (i.e., the agreement with the anatomical and temporal constraints) depends on the rate of change from randomness towards determinism. The annealing schedule defines the rate in terms of the time-dependent temperature *T*(*n*):
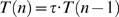
where *n* is the iteration step of the algorithm and *τ* is the annealing factor.

By allowing changes that reduce fit during early iterations of the algorithm while later making these changes very improbable, the use of temperature allows the algorithm to avoid local minima, i.e., solutions that fulfill a subset of the constraints but are not the optimal solution. Other gradient descent algorithms could have gotten “stuck” in these local minima because multiple connections need to be flipped to result in an increase of the fitness.

The employed method makes it possible to generate networks that fit both timing and connectivity data without any need for assumptions about hierarchy or connectivity.

Figure 3 presents a schematic overview of our method. The latency data (shown on the left) and the anatomical data (shown on the right) are transformed into constraints. Starting from a random connectivity pattern, the simulated annealing algorithm (shown in the middle) uses both types of constraints to generate candidate connectivity patterns that obey the constraints. Our method can be used with any selection of brain areas and can easily be extended to include newer data as it becomes available. Other kinds of data can also be added with relative ease as long as a fitness function can be devised for it.

### Parameters

We used the following parameters for the optimization algorithm in all our simulations. The initial temperature was *T*(0) = 4 and the annealing factor was set to *τ* = 0.99, the maximum number of annealing iterations was set to 1500, and the parameter *α* was set to 0.5 so the contribution of timing (*f*_lat_) and connectivity (*f*_anat_) fit to the total fitness was balanced. It should be noted that the two measures of fit, *f*_anat_ and *f*_lat_, are not necessarily equally sensitive to changing the state of one connection. For every solution we ensured that when the algorithm terminates, at least hundred iterations of the simulated annealing algorithm had not lead to new solutions.

We repeated all our simulations to show that the results are stable over a wide range of values for the above parameters. We varied the values of *α*, *T*(0), *τ*. We also varied the amount of connections in the randomly generated network that serves as the starting point for the annealing algorithm (initial edge density). For *α* and initial edge density the range could be varied over the entire possible range. The results of these tests can be seen in Figure 10. Except for the drop-offs at extreme values of *α* and a few small peaks, the results are essentially stable over the entire range of the parameters. The drop-offs are a result of the fact that at extreme values of *α*, the networks are fitted to one fit function only (either *f*_lat_ or *f*_anat_), instead of to two. The small peaks are sub-optimal solutions that disappear after the averaging of results described in the next section.

### Analysis of the Results

Because the optimization method described above is stochastic, some form of averaging over multiple solutions is needed. In our method, multiple solutions are multiple connection matrices. All our results are based on 1000 computed optimal connection matrices, each using different initial connection matrices. All the matrices can be summarized into one matrix by defining each element as a probability of the presence of a connection. When a number of solutions are generated by our method, two types of values will be found in the cells. The first type does not change when increasing the number of solutions, and these values are always 100 or 0 percent. The values of 100 and 0 percent represent connections that were present or did not exist, respectively, in all the simulations. The second type has a value that asymptotically moves closer to an intermediate percentage. For instance, the value of 50 percent means that this particular connection is present in half of the solutions and, presumably, does not matter for the fit of this network. These connections are connections that were not constrained by our data; they have not been researched according to CoCoMac [15] and do not matter for the first spike timing in the network (e.g., they could represent a feedback connection instead of a feedforward connection). When we excluded all the solutions that are not optimal (as expressed by the amount of total fit with the data) all elements have values of 0, 50, or 100 percent. The resulting connection matrices therefore only contain the values 0 (0%) 1 (100%) or they will be empty (50%).

The computational tools needed to import the data from the CoCoMac database (Cocomac Import/Export Tool) and the tools to perform the optimization and analyze the data (BrainAnnealer) are custom programs written in Object Pascal with the Borland Delphi compiler. Tools, source and documentation together with the data used can be downloaded at http://www.capalbo.nl.
